# Alcohol consumption, cigarette smoking and the risk of subtypes of head-neck cancer: results from the Netherlands Cohort Study

**DOI:** 10.1186/1471-2407-14-187

**Published:** 2014-03-14

**Authors:** Denise HE Maasland, Piet A van den Brandt, Bernd Kremer, R Alexandra (Sandra) Goldbohm, Leo J Schouten

**Affiliations:** 1 Department of Epidemiology, GROW - School for Oncology & Developmental Biology, Maastricht University, P.O. Box 616 Maastricht 6200, MD, The Netherlands; 2 Department of Otorhinolaryngology, Head & Neck Surgery, GROW - School for Oncology & Developmental Biology, Maastricht University Medical Center, Maastricht, The Netherlands; 3 TNO, Leiden, The Netherlands

**Keywords:** Alcohol consumption, Cigarette smoking, Cohort studies, Etiology, Head-neck cancer, Head-neck cancer subtypes

## Abstract

**Background:**

Prospective data on alcohol consumption, cigarette smoking and risk of head-neck cancer (HNC) subtypes, i.e. oral cavity cancer (OCC), oro-/hypopharyngeal cancer (OHPC), and laryngeal cancer (LC), are limited. We investigated these associations within the second largest prospective study on this topic so far, the Netherlands Cohort Study.

**Methods:**

120,852 participants completed a questionnaire on diet and other cancer risk factors in 1986. After 17.3 years of follow-up, 395 HNC (110 OCC, 83 OHPC, and 199 LC) cases and 4288 subcohort members were available for case-cohort analysis using Cox proportional hazards models.

**Results:**

For total HNC, the multivariable adjusted incidence rate ratio (RR) was 2.74 (95% confidence interval (CI) 1.85-4.06) for those drinking ≥30 g ethanol/day compared with abstainers; in subtypes, RRs were 6.39 for OCC, 3.52 for OHPC, and 1.54 for LC. Compared with never cigarette smokers, current cigarette smokers had a RR of 4.49 (95%CI 3.11-6.48) for HNC overall, and 2.11 for OCC, 8.53 for OHPC, and 8.07 for LC. A significant, positive, multiplicative interaction between categories of alcohol consumption and cigarette smoking was found for HNC overall (*P* interaction 0.03).

**Conclusions:**

Alcohol consumption and cigarette smoking were independently associated with risk of HNC overall, with a positive, multiplicative interaction. The strength of these associations differed among HNC-subtypes: OCC was most strongly associated with alcohol consumption but most weakly with cigarette smoking, whereas LC was not statistically significantly associated with alcohol consumption.

## Background

Head and neck cancer (HNC) includes several malignancies that originate in the paranasal sinuses, nasal cavity, salivary glands, oral cavity, pharynx, and larynx [1]. HNC is the seventh most common type of cancer in the world and in the European Union; in Europe, HNC accounts for an estimated 130,000 new cases every year [2].

Alcohol consumption and cigarette smoking are established risk factors for HNC originating from the oral cavity, pharynx, and larynx, and are likely to be differentially associated with risk of those HNC-subtypes [3-8]. However, the majority of conducted studies are case-control studies, a study design susceptible to misclassification with regard to exposure. Prospective cohort studies are less sensitive to this bias, but only six population-based cohort studies have reported on alcohol consumption, cigarette smoking and HNC-risk [9-15]. Of these studies, most had a small number of cases and were thereby hardly able to examine subtypes; HNC was often combined with other cancers into upper aerodigestive tract cancer [9,12-15]. In addition, the largest prospective study so far lacked information on smoking duration [10]. Finally, a greater than multiplicative joint effect between alcohol and tobacco consumption has been shown, but most evidence comes from case-control studies as well [9,12-14,16-18].

Therefore, we wanted to investigate these associations in HNC-subtypes within the large prospective Netherlands Cohort Study (NLCS). We focused on the most frequent HNC-subtypes: those located in the oral cavity, pharynx, and larynx, and hypothesized that 1) alcohol consumption and cigarette smoking are strongly, positively associated with HNC-risk, with multiplicative interaction, and that 2) these risks are different for oral cavity cancer (OCC), oro-/hypopharyngeal cancer (OHPC), and laryngeal cancer (LC).

## Methods

### Design and study population

The present study was conducted within the NLCS, which started in September 1986 with the inclusion of 120,852 participants, aged 55-69 years from 204 Dutch municipal population registries [19].

For data processing and analysis, the case-cohort design was used for reasons of efficiency [20]. Cases were derived from the total cohort, whereas the number of person-years at risk for the total cohort was estimated from a subcohort of 5000 persons, randomly sampled from the entire cohort at baseline.

Follow-up for cancer incidence was done by annual record linkage to the Netherlands Cancer Registry and the nationwide network and pathology registry [21]. The completeness of cancer follow-up is estimated to be ≥96% [22], and follow-up for vital status of the subcohort was nearly 100% complete after 17.3 years.

We excluded cohort members who reported to have prevalent cancer other than skin cancer at baseline, and cases and subcohort members with missing data on exposure or confounding variables. Only microscopically confirmed, first occurrences of squamous cell carcinomas – which include nearly all malignancies of the mouth, pharynx, and larynx [1,3] – of the head and neck were included.

**Figure 1:**
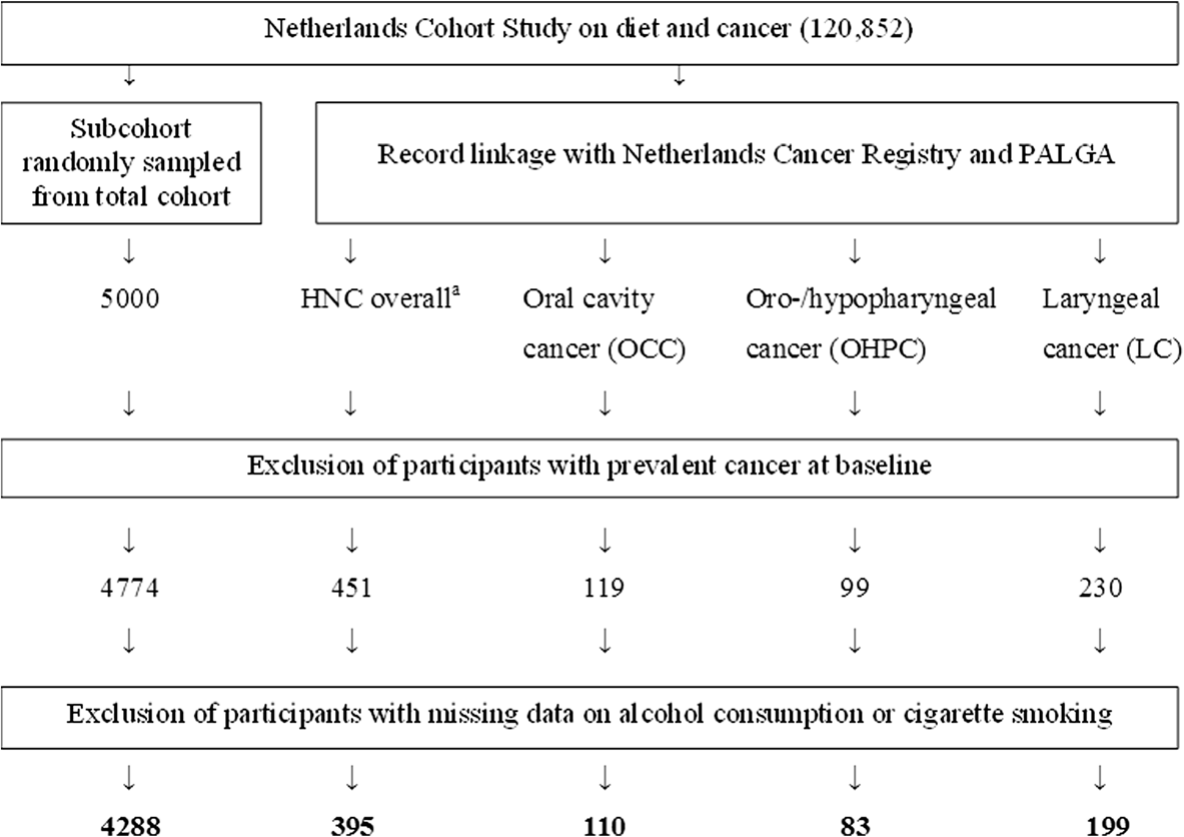
**Flow diagram of the number of subcohort members and cases on whom the analyses were based.** *PALGA: nationwide network and registry of histopathology and cytopathology in the Netherlands. ^a^Oral cavity cancer; oro-/hypopharyngeal cancer; oral cavity, pharynx unspecified or overlapping cancer; laryngeal cancer.

In total, 395 incident HNC cases and 4288 subcohort members were available for analysis (Figure 1). Of these cases, 110 were oral cavity cancer (ICD-O-3 C003-009, C020-C023, C030-C031, C039-C041, C048-C050, C060-C062, C068-C069), 83 oro-/hypopharyngeal cancer (C019, C024, C051-C052, C090-C091, C098-C104, C108-C109, C129-C132, C138-C139); 3 oral cavity, pharynx unspecified, or overlapping (C028-C029, C058-C059, C140-C142, C148), and 199 laryngeal cancer (C320-C329) cases, classified as proposed by Hashibe et al. [23], according to the International Classification of Diseases for Oncology (ICD-O-3) [24].

The NLCS has been approved by the Medical Ethics Committee of Maastricht University (Maastricht, The Netherlands).

### Exposure information

At baseline, all cohort members completed a self-administered questionnaire, which included a 150-item food frequency questionnaire (FFQ) with detailed questions on alcohol consumption, smoking habits, and other cancer risk factors.

We asked about the habitual intake of alcohol during the year preceding the start of the study, measured by six items: (1) beer; (2) red wine; (3) white wine; (4) sherry and other fortified wines; (5) liquor types containing on average 16% alcohol; and (6) (Dutch) gin, brandy, and whisky. In addition, questions were asked about the frequency of consumption and the number of glasses consumed on each drinking occasion. For analysis, we combined (2), (3), and (4) into “wine”, and (5) and (6) into “liquor”. Total mean daily ethanol intake was calculated using the Dutch food-composition table [25]. On the basis of pilot study data, standard glass sizes were defined as 200 mL for beer, 105 mL for wine, and 45 mL for liquor, corresponding to 8g, 10g, and 13g of ethanol, respectively [26]. We also asked questions about the consumption of “beer” and “other alcoholic beverages” 5 years before baseline and selected participants with stable alcohol consumption to perform a sensitivity analysis [27]. Participants who indicated that they used alcoholic beverages never or less than once a month were considered abstainers.

We asked detailed information regarding cigarette smoking. Among others, questions were asked about whether the subject was a smoker at baseline; age at which they started and stopped smoking; the number of cigarettes smoked daily and the number of smoking years (excluding stopping periods). Based on these questions, the following variables were constructed for analysis: smoking status (never/former/current); current smoking (yes/no); frequency (cigarettes/day); duration (years); the number of pack-years; and time since smoking cessation (years). We also asked about cigar and pipe smoking and the use of smokeless tobacco. Participants who indicated they had never smoked cigarettes were considered never smokers.

The FFQ was validated against a 9-day diet record, and the Spearman correlation coefficient between the alcohol intake assessed by the questionnaire and that estimated by the diet record was 0.89 for all subjects and 0.85 for users of alcoholic beverages [28]. The reproducibility of the FFQ was assessed through annually repeated measurements in a subgroup of the subcohort and the test-retest correlation was 0.90 for alcohol intake; this correlation declined only 0.01-0.02 per year [29].

Data were key-entered and processed in a standardized manner, blinded with regard to case/subcohort status in order to minimize observer bias in coding and data interpretation.

### Data analysis

Person-years at risk were calculated from baseline until diagnosis of HNC, death, emigration, loss to follow-up or end of follow-up (i.e. 31 December 2003), whichever occurred first.

Age (years) and sex were considered predefined confounders. The potential confounders considered were [3,30,31]: level of education, non-occupational physical activity, energy intake, coffee and tea consumption, intake of fruit, vegetables, fish, fat, red meat, meat products, and family history of head-neck cancer. Alcohol consumption and cigarette smoking were mutually adjusted in statistical models. A variable was considered a confounder if including it in the model changed the rate ratio (RR) for any of the cancer (sub-) types by >10%; according to this, none of the potential confounders was included in the final model.

The Cox proportional hazards model was used to estimate incidence RRs and corresponding 95% confidence intervals (CI) for alcohol consumption and cigarette smoking in multivariable adjusted case-cohort analyses. Analyses were done using the Stata 11.2 statistical software package (StataCorp, College Station, Texas, USA). Standard errors were calculated using the robust Huber-White sandwich estimator to account for additional variance introduced by sampling from the cohort; this method is equivalent to the variance-covariance estimator by Barlow [32]. The proportional hazards (PH) assumption was assessed using the scaled Schoenfeld residuals [33]. If there was an indication for violation of the assumption for a variable, we further investigated this by adding a time-varying covariate for that variable to the model.

We also analyzed beer, wine, and liquor consumption, adjusted for ethanol intake, to examine whether substances in alcoholic beverages, other than ethanol, have an effect on HNC-risk. In smoking analyses, different aspects of cigarette smoking were investigated and mutually adjusted for, in order to obtain a complete exposure model. The total number of cases that exclusively smoked cigar and/or pipe or used smokeless tobacco was too low (*N* < 10) to further analyze associations with HNC-risk.

When adjusting for smoking frequency, duration, or pack-years, we centered these continuous variables as proposed by Leffondré et al. [34]

Tests for linear dose-response trends were assessed by fitting ordinal exposure variables as continuous terms. To evaluate possible multiplicative interaction between categories of alcohol consumption and cigarette smoking, we estimated RRs of HNC overall and all HNC-subtypes for combinations of these exposures. The interaction was investigated by including cross-product terms in the model and performing a Wald test. Two-sided *P* values are reported throughout the article.

Tests for heterogeneity among HNC-subtypes were performed to investigate differences between HNC subtypes by a bootstrapping method developed for the case-cohort design [35]. For each bootstrap sample, X subcohort members were randomly drawn from the subcohort of X subjects and Y cases from the total of Y cases outside the subcohort, both with replacement, out of the dataset of X + Y observations. The logHRs were obtained from this sample using Stata’s competing risks procedure and recalculated for each bootstrap-replication. The confidence interval and *P* value of the differences in hazard ratio of the subtypes were then calculated from the replicated statistics. Each bootstrap analysis was based on at least 1,000 replications [36].

## Results

Compared to the subcohort, cases were more frequently men than women, and less often alcohol abstainers (Table 1). Among alcohol consumers, cases had a substantially higher alcohol intake and generally drank more beer, wine, and liquor than subcohort members. In both cases and subcohort members, men mostly consumed beer and liquor, whereas women drank more wine. With respect to cigarette smoking, cases were far more often current smokers and also smoked a substantially higher number of pack-years than subcohort members. Women were more often never smokers than men; among ever smokers, men generally smoked more pack-years than women, in cases and subcohort members.

**Table 1:** **Characteristics of cases and subcohort members in the Netherlands Cohort Study** (**NLCS**), **1986** - **2003**

	**Subcohort**	**Head**-**neck cancer cases**			
		**Overall**	**Subtypes**		
			**OCC**^**a**^	**OHPC**^**a**^	**LC**^**a**^
**Exposure variables and potential confounders**	**( *N * = 4288)**^**b**^	**( *N * = 395)**^**b**^	**(*N*** **= 110)**^**b**^	**(*N*** **= 83)**^**b**^	**(*N*** **= 199)**^**b**^
Age at baseline (years)	61.3 (4.2)^c^	61.8 (4.1)	61.8 (4.3)	61.5 (4.2)	61.8 (4.0)
Sex: men (%)	49.2	79.5	59.1	73.5	94.0
Abstainer from alcohol (%)	23.9	12.4	10.9	13.3	13.1
Men (%)	14.8	9.2	4.6	9.8	10.7
Women (%)	32.6	24.7	20.0	22.7^d^	50.0^e^
Alcohol consumers:					
Ethanol intake (grams/day)	13.4 (15.0)	27.3 (25.6)	28.7 (25.4)	35.0 (31.6)	23.2 (22.1)
Men	17.5	29.1	34.3	40.2	23.5
Women	8.5	18.6	19.1	18.0	14.7
Beer intake (glasses/week)	2.2 (5.8)	6.3 (12.5)	4.4 (10.2)	10.3 (18.3)	5.8 (10.4)
Men	3.7	6.9	6.1	11.1	5.8
Women	0.3	3.5	1.4	7.9	4.5
Wine intake (glasses/week)	3.7 (5.6)	4.4 (9.1)	5.5 (8.1)	6.0 (11.5)	3.1 (8.4)
Men	3.1	4.0	4.2	6.7	3.1
Women	4.4	6.0	7.7	3.5	4.1
Liquor intake (glasses/week)	2.9 (5.7)	7.3 (10.0)	8.4 (11.2)	7.7 (11.7)	6.4 (8.3)
Men	4.6	8.2	11.5	9.4	6.6
Women	0.9	3.0	3.1	1.9	1.9
Cigarette smoking status					
Total					
Never smokers (%)	36.9	11.1	26.4	7.2	4.5
Former smokers (%)	35.5	27.9	21.8	26.5	32.2
Current smokers (%)	27.6	61.0	51.8	66.3	63.3
Men					
Never smokers (%)	13.8	6.7	16.9	4.9	3.7
Former smokers (%)	51.4	31.5	27.7	29.5	33.7
Current smokers (%)	34.8	61.8	55.4	65.6	62.6
Women					
Never smokers (%)	59.4	28.4	40.0	13.6^d^	16.7^e^
Former smokers (%)	20.0	13.6	13.3	18.2	8.3
Current smokers (%)	20.6	58.0	46.7	68.2	75.0
Ever cigarette smokers:					
Frequency of cigarette smoking (*N*/day)	15.3 (10.2)	19.5 (10.9)	19.9 (11.9)	21.4 (12.7)	18.6 (9.6)
Men	17.1	19.9	21.6	22.9	18.4
Women	11.6	17.4	16.5	16.7	22
Duration of cigarette smoking (years)	31.7 (12.1)	39.1 (9.5)	37.8 (9.2)	38.5 (9.8)	39.9 (9.4)
Men	33.6	39.8	38.9	39.8	40.0
Women	27.8	35.8	35.4	34.5	38.2
Pack-years of cigarette smoking (*N*)	22.8 (17.7)	34.4 (20.9)	34.5 (23.5)	37.0 (23.2)	33.5 (18.9)
Men	26.2	35.7	38.5	40.5	33.2
Women	16.0	28.3	26.4	26.3	38.5
Level of education (%)					
Primary	29.5	27.6	20.9	25.6	31.5
Lower vocational	22.0	18.9	17.3	17.1	20.8
Secondary and medium vocational	34.8	35.7	41.8	36.6	32.5
University and higher vocational	13.8	17.9	20.0	20.7	15.2

^a^OCC: oral cavity cancer; OHPC: oro-/hypopharyngeal cancer; LC: laryngeal cancer.

^b^The number of subcohort members or cases used in age- and sex-adjusted, multivariate analyses of alcohol consumption and cigarette smoking.

^c^Values are given as mean (SD); for categorical variables, *N* (%) is presented.

^d^Based on only 22 female OHPC cases.

^e^Based on only 12 female LC cases.

### Alcohol consumption

Alcohol consumption of ≥30 grams (g) per day compared with abstinence was associated with a statistically significantly increased risk of HNC overall (multivariate RR = 2.74, 95% CI 1.85-4.06), OCC (RR = 6.39, 95% CI 3.13-13.03), and OHPC (RR = 3.52, 95% CI 1.69-7.36), but not LC (RR = 1.54, 95% CI 0.91-2.60) (Table 2). A strong dose-response relationship (*P* trend < 0.001) was found between categories of increasing alcohol consumption and HNC overall, OCC, and OHPC risk. A significant interaction was found between sex and continuous alcohol consumption in HNC overall (*P* = 0.02) and OCC (*P* = 0.004), with women having higher RRs than men.

**Table 2:** **Associations** (**multivariable**^**a **^**adjusted incidence RRs**) **between alcohol consumption and risk of subtypes of head**-**neck cancer**; **Netherlands Cohort Study** (**NLCS**), **1986** – **2003**

		**Subcohort**	**Head**-**neck cancer cases**								
			**Overall**		**Subtypes**						
					**OCC**^**b**^		**OHPC**^**b**^		**LC**^**b**^		
	**Categorical median**	**Person time at risk (years)**	**No. of cases**	**RR (95% CI)**	**No. of cases**	**RR (95% CI)**	**No. of cases**	**RR (95% CI)**	**No. of cases**	**RR (95% CI)**	***P *** **for heterogeneity**
**Alcohol consumption (grams ethanol/day)**											
Abstainers	0	15 255	49	1 (reference)	12	1 (reference)	11	1 (reference)	26	1 (reference)	0.86
>0 to <5	2	19 008	67	1.11 (0.75-1.65)	17	1.25 (0.59-2.65)	14	1.06 (0.47-2.40)	36	1.03 (0.60-1.77)	
5 to <15	9	14 468	72	1.15 (0.77-1.71)	19	1.91 (0.91-4.03)	12	0.90 (0.38-2.13)	40	0.94 (0.56-1.58)	
15 to <30	22	9 961	92	1.52 (1.02-2.27)	30	3.88 (1.86-8.12)	13	0.99 (0.41-2.38)	49	1.10 (0.66-1.83)	
≥30	40	5 659	115	2.74 (1.85-4.06)	32	6.39 (3.13-13.03)	33	3.52 (1.69-7.36)	48	1.54 (0.91-2.60)	
*P* for trend^c^				<0.001		<0.001		<0.001		0.05	
Continuous, 10 gram											
ethanol/day increments											
Overall		64 352	395	1.20 (1.12-1.27)	110	1.28 (1.18-1.39)	83	1.27 (1.16-1.38)	199	1.10 (1.02-1.18)	0.18
Men		30 169	314	1.19 (1.12-1.27)	65	1.27 (1.17-1.38)	61	1.27 (1.16-1.39)	187	1.10 (1.03-1.19)	
Women		34 183	81	1.40 (1.18-1.65)	45	1.58 (1.33-1.87)	22	1.31 (0.91-1.87)	12	0.85 (0.46-1.59)	
*P* for interaction^d^				0.02		0.004		0.68		0.67	
**Alcohol consumption (grams ethanol/day) stable users**^**e**^											
Abstainers	0	11 810	38	1 (reference)	9	1 (reference)	9	1 (reference)	20	1 (reference)	1.00
>0 to <5	2	11 813	36	0.98 (0.60-1.61)	12	1.65 (0.68-4.01)	7	0.86 (0.30-2.41)	17	0.72 (0.35-1.46)	
5 to <15	9	8 749	38	0.96 (0.58-1.59)	9	1.68 (0.63-4.47)	8	0.89 (0.32-2.47)	21	0.72 (0.37-1.40)	
15 to <30	22	5 293	45	1.27 (0.76-2.11)	12	3.20 (1.25-8.19)	6	0.72 (0.23-2.26)	27	0.96 (0.50-1.83)	
≥30	42	3 047	69	2.90 (1.78-4.73)	17	7.50 (3.15-17.88)	20	3.46 (1.46-8.20)	31	1.57 (0.82-3.02)	
*P* for trend				<0.001		<0.001		0.001		0.03	
Continuous, 10 grams		39712	226	1.26 (1.16-1.36)	59	1.37 (1.24-1.52)	50	1.35 (1.20-1.52)	116	1.16 (1.04-1.28)	0.72
ethanol/day increments											
**Alcoholic beverages (glasses/day)**^**f**^											
**Beer**											
No beer	0	43 519	183	1 (reference)	59	1 (reference)	36	1 (reference)	87	1 (reference)	0.84
>0- < 1	0.2	16 408	129	0.94 (0.71-1.24)	34	1.10 (0.65-1.86)	24	0.98 (0.54-1.76)	69	0.85 (0.60-1.22)	
1- < 2	1.4	2 853	37	1.12 (0.72-1.74)	8	1.17 (0.49-2.77)	6	1.04 (0.41-2.66)	23	1.19 (0.71-2.01)	
≥2	3.4	1 554	46	1.39 (0.83-2.34)	9	0.99 (0.34-2.82)	17	2.48 (1.03-5.98)	20	1.30 (0.69-2.46)	
*P* for trend				0.14		0.95		0.03		0.20	
Continuous, 1		64 335	395	1.07 (0.97-1.19)	110	0.97 (0.80-1.16)^g^	83	1.19 (1.01-1.40)	199	1.08 (0.96-1.23)	0.07
glass/day increments											
**Wine**											
No wine	0	30 263	197	1 (reference)	44	1 (reference)	38	1 (reference)	114	1 (reference)	0.93
>0- < 1	0.2	25 975	132	0.88 (0.67-1.14)	40	1.07 (0.67-1.71)	33	1.01 (0.59-1.75)	57	0.74 (0.52-1.05)	
1- < 2	1.4	5 277	39	0.95 (0.63-1.44)	14	1.31 (0.67-2.55)	5	0.52 (0.19-1.39)	20	1.07 (0.63-1.83)	
≥2	2.6	2 751	24	0.56 (0.29-1.07)	11	0.93 (0.34-2.57)	7	0.52 (0.15-1.81)^g^	6	0.39 (0.15-0.99)	
*P* for trend				0.15		0.93		0.16		0.21	
Continuous, 1		64 265	392	0.88 (0.74-1.05)	109	0.89 (0.69-1.16)	83	0.86 (0.64-1.17)	197	0.88 (0.68-1.14)	0.26
glass/day increments											
**Liquor**											
No liquor	0	33 299	137	1 (reference)	40	1 (reference)	34	1 (reference)	63	1 (reference)	0.44
>0- < 1	0.2	23 492	133	1.09 (0.84-1.43)	31	1.10 (0.67-1.80)	23	0.86 (0.48-1.53)	78	1.17 (0.81-1.67)	
1- < 2	1.9	5 370	67	1.09 (0.76-1.57)	18	1.65 (0.87-3.15)	12	0.79 (0.39-1.62)	37	1.08 (0.67-1.74)	
≥2	2.8	2 115	56	1.18 (0.71-1.95)	20	2.26 (1.02-4.99)	14	0.83 (0.33-2.13)	20	0.95 (0.47-1.93)	
*P* for trend				0.61		0.03		0.64		0.83	
Continuous, 1		64 275	393	1.01 (0.86-1.18)	109	1.18 (0.89-1.56)	83	0.89 (0.68-1.15)	198	0.98 (0.80-1.21)	0.25
glass/day increments											

^a^Adjusted for age (years), sex, cigarette smoking (status (never/former/current), frequency (continuous; centered), and duration (continuous; centered)).

^b^OCC: oral cavity cancer; OHPC: oro-/hypopharyngeal cancer; LC: laryngeal cancer.

^c^Tests for dose-response trends were assessed by fitting ordinal variables as continuous terms in the Cox proportional hazards model.

^d^*P* Value for interaction between sex and alcohol consumption, based on cross-product terms in the Cox proportional hazards model and Wald test.

^e^Subjects who had not changed their continuous alcohol consumption habits in the 5 years before baseline: for “beer” and “other alcoholic beverages”, participants could indicate whether 5 years before baseline they drunk (1) more than, (2) equal amounts of or (3) less than at baseline; the fourth answer option was (4) “I never use this”.

^f^Additionally adjusted for continuous ethanol intake (g ethanol/day).

^g^Proportional hazards assumption was possibly violated for the exposure variable, and there was a statistically significant interaction with time.

After adjustment for total alcohol intake, consumption of beer, wine, and liquor was generally not significantly associated with HNC-risk. Beer consumption was, however, statistically significantly, positively associated with OHPC-risk (*P* trend = 0.03); liquor consumption was significantly associated with an increased risk of OCC (*P* trend = 0.03). Wine consumption was largely inversely associated – although not statistically significantly – with risk of HNC overall and HNC-subtypes.

Although risk rates clearly varied among HNC-subtypes, tests for heterogeneity did not show any significant risk differences, possibly due to low power.

### Cigarette smoking

Current cigarette smoking was statistically significantly associated with risk of HNC overall (multivariate RR = 4.49, 95% CI 3.11-6.48) and all subtypes, with strongest associations in OHPC (RR = 8.53, 95% CI 3.38-21.55) and LC (RR = 8.07, 95% CI 3.94-16.54), compared with never smoking (Table 3). Compared with never smoking, former cigarette smoking was also associated with risk of HNC overall, although not statistically significantly (RR = 1.44, 95% CI 0.97-2.14), OHPC (RR = 2.68, 95% CI 1.00-7.14), and LC (RR = 2.63, 95% CI 1.26-5.47), but not OCC (RR = 0.70, 95% CI 0.37-1.33). Frequency and duration of cigarette smoking were also strongly, statistically significantly associated with an increased risk of HNC overall, OHPC, and LC (Table 3).

**Table 3:** **Associations** (**multivariable**^**a **^**adjusted incidence RRs**) **between cigarette smoking and risk of subtypes of head**-**neck cancer**; **Netherlands Cohort Study** (**NLCS**), **1986** - **2003**

		**Subcohort**	**Head**-**neck cancer cases**								
			**Overall**		**Subtypes**						
					**OCC**^**b**^		**OHPC**^**b**^		**LC**^**b**^		
	**Categorical median**	**Person time at risk (years)**	**No. of cases**	**RR (95% CI)**	**No. of cases**	**RR (95% CI)**	**No. of cases**	**RR (95% CI)**	**No. of cases**	**RR (95% CI)**	***P *** **for heterogeneity**
**Cigarette smoking status**											
Never smokers		25051	44	1 (reference)	29	1 (reference)	6	1 (reference)	9	1 (reference)	0.97
Former smokers		22644	110	1.44 (0.97-2.14)	24	0.70 (0.37-1.33)	22	2.68 (1.00-7.14)^i^	64	2.63 (1.26-5.47)	
Current smokers		16657	241	4.49 (3.11-6.48)	57	2.11 (1.23-3.62)	55	8.53 (3.38-21.55)	126	8.07 (3.94-16.54)	
*P* for trend^c^				<0.001		0.001		<0.001		<0.001	
*P* for interaction with sex^d^				0.25		0.08		0.44		0.46	
**Cigarette smoking status, additionally adjusted for frequency and duration of cigarette smoking**^**e**^											
Never smokers		25051	44	1 (reference)	29	1 (reference)	6	1 (reference)	9	1 (reference)	0.97
Former smokers		22644	110	1.64 (1.08-2.49)	24	0.79 (0.40-1.58)	22	3.03 (1.09-8.45)	64	2.87 (1.34-6.13)	
Current smokers		16657	241	3.51 (2.36-5.23)	57	1.91 (1.06-3.42)	55	7.49 (2.87-19.54)	126	5.26 (2.45-11.28)	
*P* for trend				<0.001		0.03		<0.001		<0.001	
**Frequency of cigarette smoking ( *N */day)**^**f**^											
Never smokers	0	25051	44	1 (reference)	29	1 (reference)	6	1 (reference)	9	1 (reference)	0.99
>0 to <20	10	24787	155	1.30 (0.84-2.01)	38	0.63 (0.30-1.32)	30	2.08 (0.73-5.94)	85	2.32 (1.07-5.04)	
≥20	20	14514	196	2.23 (1.45-3.44)	43	1.06 (0.52-2.16)	47	4.67 (1.64-13.34)	105	3.75 (1.73-8.14)	
*P* for trend				<0.001		0.33		<0.001		<0.001	
Continuous, 10 cigarettes/day increments		64352	395	1.25 (1.13-1.38)	110	1.20 (1.00-1.44)^j^	83	1.42 (1.20-1.69)	199	1.21 (1.08-1.36)	0.71
**Duration of cigarette smoking (years)**^**g**^											
Never smokers	0	25051	44	1 (reference)	29	1 (reference)	6	1 (reference)	9	1 (reference)	<0.001
>0 to <20	13	7433	20	1.00 (0.56-1.77)	4	0.38 (0.13-1.17)	5	2.11 (0.59-7.51)	11	1.88 (0.75-4.69)	
20 to <40	30	18999	105	1.44 (0.95-2.21)	30	0.80 (0.41-1.59)	25	2.74 (0.98-7.68)	50	2.35 (1.09-5.06)	
≥40	43	12868	226	2.45 (1.49-4.02)	47	0.98 (0.39-2.46)	47	3.89 (1.22-12.40)	129	4.81 (2.11-11.00)	
*P* for trend				<0.001		0.87		0.02		<0.001	
Continuous, 10 years increments		64352	395	1.28 (1.14-1.42)	110	1.03 (0.85-1.24)	83	1.36 (1.09-1.70)	199	1.49 (1.25-1.78)	0.25
**Pack-years of cigarette smoking**^**h**^											
Never smokers	0	25051	44	1 (reference)	29	1 (reference)	6	1 (reference)	9	1 (reference)	1.00
>0 to <20	9	20832	96	1.16 (0.77-1.76)	24	0.58 (0.30-1.14)	20	2.12 (0.77-5.80)	51	2.07 (0.97-4.41)	
20 to <40	28	12732	132	1.65 (1.04- 2.60)	32	0.84 (0.39-1.83)	26	2.87 (0.94-8.79)	73	2.93 (1.33-6.48)	
≥40	48	5736	123	2.82 (1.76-4.50)	25	1.28 (0.58-2.82)	31	6.49 (2.11-19.95)	66	4.79 (2.15-10.64)	
*P* for trend				<0.001		0.07		<0.001		<0.001	
Continuous, 10 pack-years increments		64352	395	1.18 (1.11-1.25)	110	1.16 (1.04-1.28)	83	1.24 (1.12-1.36)	199	1.16 (1.09-1.24)	0.77
**Cigarette smoking cessation**^**i**^											
Never smokers	25	25051	44	1 (reference)	29	1 (reference)	6	1 (reference)	9	1 (reference)	<0.001
Stopped ≥20 years	14	6953	24	1.25 (0.72-2.19)	5	0.63 (0.22-1.81)	6	3.35 (0.97-11.55)	13	1.92 (0.79-4.70)	
Stopped 10 to <20 years	5	7717	36	1.49 (0.91-2.43)	8	0.78 (0.32-1.86)	8	3.29 (1.04-10.39)	20	2.45 (1.07-5.61)^k^	
Stopped >0 to <10 years	0	7918	50	1.73 (1.09-2.76)	11	0.84 (0.39-1.83)	8	2.48 (0.77-7.93)	31	3.45 (1.56-7.62)	
Current smokers		16657	241	4.26 (2.93-6.20)	57	2.03 (1.16-3.56)	55	8.10 (3.14-20.87)	126	7.53 (3.65-15.51)	
*P* for trend				<0.001		0.004		<0.001		<0.001	

^a^All analyses were adjusted for age (years), sex, and alcohol consumption (g ethanol/day; continuous).

^b^OCC: oral cavity cancer; OHPC: oro-/hypopharyngeal cancer; LC: laryngeal cancer.

^c^Tests for dose-response trends were assessed by fitting ordinal variables as continuous terms in the Cox proportional hazards model.

^d^*P* Value for interaction between sex and cigarette smoking status, based on cross-product terms in the Cox proportional hazards model and Wald test.

^e^Additionally adjusted for frequency (*N*/day; continuous; centered) and duration of cigarette smoking (years; continuous; centered).

^f^Analyses of cigarette smoking frequency were additionally adjusted for current cigarette smoking and duration of cigarette smoking (years; continuous; centered).

^g^Analyses of cigarette smoking duration were additionally adjusted for current cigarette smoking and frequency of cigarette smoking (*N*/day; continuous; centered).

^h^Analyses of cigarette smoking pack-years were additionally adjusted for current cigarette smoking.

^i^Cigarette smoking cessation was additionally adjusted for the no. of cigarette pack-years (continuous; centered).

^j^*P* < 0.05.

^k^Proportional hazards assumption was possibly violated for the exposure variable, and there was a statistically significant interaction with time.

Regarding different aspects of cigarette smoking, after mutual adjustment, cigarette smoking status, frequency, and duration all remained statistically significantly associated with risk of HNC overall, OHPC, and LC (see Additional file 1). After additional adjustment for alcohol consumption (Table 3), most RRs between cigarette smoking status, frequency, duration and risk of HNC(-subtypes) slightly attenuated, but remained statistically significantly associated with increased risks.

Results regarding smoking cessation show that the risk of HNC overall and all subtypes diminished for smokers who stopped smoking since <10, 10 to <20, or ≥20 years, compared with current smokers (all *P* trend < 0.01) (Table 3). Nevertheless, compared with never smokers, RRs 20 years after smoking cessation were still elevated for HNC overall, OHPC, and LC, although not statistically significantly.

Despite considerable differences in risk rates among HNC-subtypes, tests for heterogeneity only showed statistically significant risk rates for duration of cigarette smoking (*P* < 0.001) and time since smoking cessation (*P* < 0.001).

### Interaction between alcohol consumption and cigarette smoking

For HNC overall, increased risks were found for every exposure combination of alcohol consumption and cigarette smoking, mostly statistically significantly, compared to never smokers and abstainers as reference group (Table 4). In addition, a statistically significant, positive, multiplicative interaction was found (*P* interaction 0.03) between categories of alcohol consumption and cigarette smoking, with a RR of 8.28 (95% CI 3.98-17.22), comparing participants smoking ≥ 20 cigarettes and drinking ≥30 g alcohol per day with never smokers abstaining from alcohol.

**Table 4:** **Combinations of categories of alcohol consumption and cigarette smoking and risk** (**multivariable**^**a **^**adjusted incidence RRs**) **of subtypes of head**-**neck cancer**; **Netherlands Cohort Study** (**NLCS**), **1986** - **2003**

	**Head**-**neck cancer cases**										
	**Overall**					**Subtypes**					
						**OCC**^**b**^		**OHPC**^**b**^		**LC**^**b**^	
	**Alcohol consumption (grams ethanol/day)**					**Alcohol consumption**^**c**^		**Alcohol consumption**^**c**^		**Alcohol consumption**^**c**^	
	**0**	**>0 to <5**	**5 to <15**	**15 to <30**	**≥30**	**0-15**	**>15**	**0-15**	**>15**	**0-15**	**>15**
**Frequency of cigarette smoking ( *N */day)**											
**Never smokers**											
Cases/person time at risk^d^	10/8959	13/9729	7/4245	11/1499	3/619	21/22933	8/2118	3/22933	3/2118	6/22933	3/2118
RR	1 (ref)	1.20	1.23	5.53	2.97	1 (ref)	4.16	1 (ref)	10.18	1 (ref)	3.05
95% CI		0.52 – 2.75	0.46 – 3.29	2.27 – 13.49	0.78 – 11.40		1.82 – 9.52		2.03 – 51.06		0.72 – 12.92
**>0 to <20**											
Cases/person time at risk	21/4194	25/6534	37/7061	40/4814	32/2184	20/17789	18/6998	18/17789	12/6998	44/17789	41/6998
RR	1.89	1.56	2.04	2.63	3.81	0.76	1.55	4.02	5.63	2.66	4.19
95% CI	0.83 – 4.34	0.71 – 3.41	0.95 – 4.40	1.22 – 5.67	1.71 – 8.51	0.34 – 1.71	0.66 – 3.62	1.07 – 15.14	1.44 – 22.00	1.03 – 6.86	1.60 – 11.02
**≥20**											
Cases/person time at risk	18/2102	29/2745	28/3162	41/3648	80/2856	7/8010	36/6504	16/8010	31/6504	52/8010	53/6504
RR	2.78	3.88	2.85	3.32	8.28	0.58	3.54	7.26	16.12	5.42	5.54
95% CI	1.18 – 6.54	1.77 – 8.49	1.28 – 6.34	1.52 – 7.25	3.98 – 17.22	0.21 – 1.58	1.66 – 7.52	1.86 – 28.44	4.31 – 60.27	2.10 – 13.98	2.15 – 14.27
*P* for interaction^e^					0.03		0.10		0.09		0.19

^a^Adjusted for age, sex, current cigarette smoking, and duration of cigarette smoking (years; continuous; centered).

^b^OCC: oral cavity cancer; OHPC: oro-/hypopharyngeal cancer; LC: laryngeal cancer.

^c^For the categorical interaction analyses in HNC-subtypes, it was necessary to aggregate categories of alcohol consumption (grams ethanol/day) in order to obtain sufficient numbers in strata.

^d^Person time at risk in years.

^e^*P* Value for interaction between categories of alcohol consumption and cigarette smoking, based on cross-product terms in the Cox proportional hazards model and Wald test.

In HNC-subtypes, RRs were mostly increased as well when comparing participants smoking ≥ 20 cigarettes and drinking >15 g alcohol per day with never smokers consuming 0 to 15g alcohol per day, with the highest RR for OHPC (RR = 16.12, 95% CI 4.31-60.27), but no significant interaction was found, possibly due to low numbers in strata.

## Discussion

In this large prospective study on alcohol consumption, cigarette smoking, and risk of HNC(-subtypes), alcohol consumption and cigarette smoking were strongly, independently associated with an increased risk of HNC overall. The strength of these associations however differed between HNC-subtypes; OCC was most strongly associated with alcohol consumption but most weakly with cigarette smoking, whereas LC was not statistically significantly associated with alcohol consumption. For HNC overall, a multiplicative interaction between categories of alcohol consumption and cigarette smoking was found.

### Alcohol consumption

Our results are in agreement with those of previous studies, showing alcohol consumption to be an independent risk factor for the development of HNC, with a strong, dose-response relationship [4,9,11-14,17,23,37,38]. Alcoholic beverages and acetaldehyde, the main metabolite of ethanol, are classified as a class I carcinogen [18]. It is plausible that alcohol – after being metabolized – acts both directly and indirectly in HNC carcinogenesis, the latter for example by acting as a solvent for other possible carcinogens, such as tobacco carcinogens [3,39].

The differential risk among HNC-subtypes is consistent with other studies, in which LC was also least associated with alcohol consumption [8,40,41]. However, several other studies found OHPC being most associated with alcohol consumption, although sometimes in specific subgroups, as opposed to OCC in our study [11,23,41]. Nevertheless, the differential risk among HNC-subtypes is likely to be explained by the larynx having the least direct exposure to alcohol compared with the oral cavity and pharynx [39,42]. The slightly increased RRs for alcohol consumption and LC may be due to inhalation of alcohol containing aerosols, silent aspiration, systemic effects, and possibly residual confounding.

After adjustment for total alcohol intake, we generally found similar risks between intake of beer, wine, liquor and HNC. These findings imply that ethanol itself probably is the most important factor in determining HNC-risk, rather than other substances in alcoholic beverages, which is in line with the results from other studies [3,11,42]. Consumption of wine was, however, generally inversely associated with HNC-risk, as was also shown in a pooled analysis [42], which could be due to residual confounding by a general healthier lifestyle of wine-consumers in our study population [3,42,43].

The significantly higher RRs between alcohol consumption and HNC risk in women as compared with men were seen earlier and could possibly be explained by women having stronger carcinogenic effects of alcohol at the same exposure level, suggesting possible gender-specific risk or protective factors [11].

### Cigarette smoking

This study confirms the strong associations of cigarette smoking with increased risk of HNC overall and all subtypes [3,5,7,10,14,23,37,41]. Among subtypes, however, OCC was least associated with cigarette smoking, and strongest associations were found with OHPC and LC. In addition, smoking status, frequency, and duration all appear to be of importance in the association between cigarette smoking and risk of HNC overall, OHPC, and LC. These results are generally consistent with previous reviews showing that cigarette smoking has a stronger effect on the larynx and/or pharynx than on the oral cavity [7,8,10,23,41]; in two meta-analyses, the larynx seemed to be clearly most susceptible to the effects of cigarette smoking [23,41]. A possible explanation for this could be the aerodynamics of respiratory flow in the upper airway: this flow changes from laminar in the oral cavity to turbulent in the larynx, which may result in the larynx and pharynx having a higher exposure to inhaled air - and thus to cigarette smoke - than the oral cavity.

Finally, our study shows smoking cessation leads to decreased HNC-risks, which is in line with results from a recent pooled analysis as well [44].

### Interaction between alcohol consumption and cigarette smoking

Our study confirms a multiplicative interaction between categories of alcohol consumption and cigarette smoking in HNC overall [9,14,16-18,37,38,41]. The interaction effect between alcohol consumption and cigarette smoking is biologically plausible, since alcohol can act as a solvent for carcinogens in cigarette smoke and make the mucosa more permeable for these carcinogens; as a result, the carcinogenic properties of both factors are likely to be enhanced in the presence of one another [3,39]. Still, in HNC-subtypes, we had low numbers of cases in strata, which probably resulted in limited power to detect a significant deviation from the multiplicative model.

### Strengths and limitations

Important strengths of our study are the prospective character and completeness of follow-up. Our study is the second largest prospective cohort study investigating alcohol consumption and cigarette smoking on the risk of HNC overall and subtypes so far [9-15]. Furthermore, we were able to take into account data on smoking duration, and to investigate as well as adjust for several aspects of smoking behavior.

A possible limitation of our study is the single measurement of exposure data. Alcohol consumption and cigarette smoking were however extensively addressed in the questionnaire, with questions about lifetime exposure history of smoking and alcohol intake 5 years before baseline. It is however possible that participants who smoked at baseline in 1986 stopped smoking at some point during follow-up or changed their alcohol intake, and this may have led to bias due to misclassification. Furthermore, although our study includes a large number of cases, a lack of power is a possible explanation for finding non-significant results for some associations and the tests for heterogeneity.

We lack information on human papillomavirus (HPV) infection. HPV-infection is associated with HNC-risk [45,46], but mainly with OHPC, in particular tonsil cancer and cancer of the base of the tongue. According to rates in our university medical center, only 25% of the diagnosed and treated oropharyngeal cancers between 1997 and 2003 were HPV-positive (all oropharyngeal cancer cases have been analyzed by p16-immunostaining and HPV16-specific fluorescence in situ hybridization (FISH), and – if FISH was negative – HPV-specific polymerase chain reaction). Moreover, the role of HPV in HNC-carcinogenesis is mainly of importance in young HNC-patients, and has increased since 1990 [47-49]. Since our participants were aged 55-69 years at baseline in 1986, we assume that the number of HPV-associated HNC cases in our cohort is low, and we expect potential bias due to possible misclassification to be very limited.

Other factors we did not take into account in our analyses are the use of drugs and oral hygiene. Although we investigated several potential confounders, residual confounding is still possible, but we presume this to be limited as well.

It might also be interesting to examine the RRs of HNC for smokers among non-drinkers and for drinkers among non-smokers. However, as the case numbers for these subgroups would be too small to analyze, we decided not to investigate this.

Finally, though we wanted to examine the role of alcohol consumption and cigarette smoking in HNC-subtypes, we did not investigate HNC located in the major salivary glands, nasal cavity, paranasal sinuses, and nasopharynx, because of low numbers of these cases as well as a presumably different etiology [50].

## Conclusions

In conclusion, the present study, which is the second largest prospective cohort study regarding this topic so far, confirms the principal role of alcohol consumption and cigarette smoking in HNC-carcinogenesis, as well as the differential associations with HNC-subtypes, and a significant, positive, multiplicative interaction between both factors. As the existing evidence is largely based on case-control studies, this cohort study contributes to establish in which extent alcohol consumption and cigarette smoking are associated with risk of HNC overall and, more specifically, HNC-subtypes.

## Abbreviations

CI: Confidence interval; FFQ: Food frequency questionnaire; FISH: Fluorescence in situ hybridization; HNC: Head and neck cancer; HPV: Human papillomavirus; LC: Laryngeal cancer; NLCS: Netherlands Cohort Study; OCC: Oral cavity cancer; OHPC: Oro-/hypopharyngeal cancer; PH: Proportional hazards; RR: Rate ratio

## Competing interests

The authors declare that they have no competing interests.

## Authors’ contributions

The Netherlands Cohort Study was set up by PAvdB and RAG. All authors participated in the analysis and interpretation of data; DHEM carried out the statistical analyses. DHEM drafted the initial manuscript, and PAvdB, BK, RAG, and LJS were involved in revising it. All authors read and approved the final manuscript. PAvdB, BK, and LJS were involved in the acquisition of funding for the study.

## Pre-publication history

The pre-publication history for this paper can be accessed here:

http://www.biomedcentral.com/1471-2407/14/187/prepub

## Supplementary Material

Additional file 1: Table A1. Age- and sex-adjusted associations (incidence RRs) between cigarette smoking and risk of subtypes of head-neck cancer; Netherlands Cohort Study (NLCS), 1986 - 2003. **Table A2.** Associations (multivariable adjusted incidence RRs) between cigarette smoking and risk of subtypes of head-neck cancer, with (mutual) adjustment for smoking aspects; Netherlands Cohort Study (NLCS), 1986 - 2003. Click here for file
