# Supplementary material for: Alcohol consumption, cigarette smoking and the risk of subtypes of head-neck cancer: results from the Netherlands Cohort Study
Source: BMC Cancer. 2014 Mar 14;14:187. doi: 10.1186/1471-2407-14-187 (PMC4004328; doi:10.1186/1471-2407-14-187)
Supplement: Additional file 1: Table A1. — Age- and sex-adjusted associations (incidence RRs) between cigarette smoking and risk of subtypes of head-neck cancer; Netherlands Cohort Study (NLCS), 1986 - 2003. Table A2. Associations (multivariable adjusted incidence RRs) between cigarette smoking and risk of subtypes of head-neck cancer, with (mutual) adjustment for smoking aspects; Netherlands Cohort Study (NLCS), 1986 - 2003. [file 1471-2407-14-187-S1.doc]

### Additional file 1

Table A1. Age- and sex-adjusted associations^a^ (incidence RRs) between cigarette smoking and risk of subtypes of head-neck cancer; Netherlands Cohort Study (NLCS), 1986 - 2003

|  |  | **Subcohort** |  | **Head-neck cancer cases** | | | | | | | | | | |
| --- | --- | --- | --- | --- | --- | --- | --- | --- | --- | --- | --- | --- | --- | --- |
|  |  |  |  | **Overall** | |  | **Subtypes** | | | | | | | |
|  |  |  |  |  | |  | OCC^b^ | |  | OHPC^b^ | |  | LC^b^ | |
|  | **Categorical median** | **Person time at risk (years)** |  | **No. of cases** | **RR**  **(95 CI%)** |  | **No. of cases** | **RR**  **(95 CI%)** |  | **No. of cases** | **RR**  **(95 CI%)** |  | **No. of cases** | **RR**  **(95 CI%)** |
| **Cigarette smoking status** | | | | | | | | | | | | | | |
| Never smokers  Former smokers  Current smokers  *P* for trend^c^ |  | 25051  22644  16657 |  | 44  110  241 | 1 (reference)  1.57 (1.07-2.31)  5.35 (3.74-7.66)  <0.001 |  | 29  24  57 | 1 (reference)  0.77 (0.41-1.44)  2.73 (1.62-4.61)  <0.001 |  | 6  22  55 | 1 (reference)  2.91 (1.09-7.71)  10.88 (4.39-26.94)  <0.001 |  | 9  64  126 | 1 (reference)  2.78 (1.34-5.77)  8.86 (4.35-18.05)  <0.001 |
| **Frequency of cigarette smoking (*N*/day)** | | | | | | | | | | | | | | |
| Never smokers  >0 to <20  ≥20  *P* for trend  Continuous, 10 cigarettes/  day increments | 0  10  20 | 25051  24787  14514  64352 |  | 44  155  196  395 | 1 (reference)  2.44 (1.68-3.55)  4.76 (3.23-7.03)  <0.001  1.39 (1.29-1.50) |  | 29  38  43  110 | 1 (reference)  1.27 (0.72-2.24)  2.48 (1.35-4.55)  0.002  1.40 (1.22-1.61) |  | 6  30  47  83 | 1 (reference)  4.42 (1.72-11.34)  11.42 (4.33-30.12)  <0.001  1.60 (1.41-1.82) |  | 9  85  105  199 | 1 (reference)  4.09 (1.98-8.44)  7.13 (3.40-14.93)  <0.001  1.29 (1.19-1.41) |
| **Duration of cigarette smoking (years)** | | | | | | | | | | | | | | |
| Never smokers  >0 to <20  20 to <40  ≥40  *P* for trend  Continuous, 10 years  increments | 0  13  30  43 | 25051  7433  18999  12868  64352 |  | 44  20  105  226  395 | 1 (reference)^d^  1.16 (0.67-2.01)  2.18 (1.46-3.26)  6.10 (4.14-9.00)  <0.001  1.60 (1.47-1.76)^e^ |  | 29  4  30  47  110 | 1 (reference)  0.47 (0.16-1.38)  1.35 (0.74-2.46)  3.01 (1.65-5.50)  <0.001  1.33 (1.15-1.53)^e^ |  | 6  5  25  47  83 | 1 (reference)  2.53 (0.75-8.55)  4.77 (1.79-12.69)  12.46 (4.77-32.53)  <0.001  1.80 (1.48-2.19) |  | 9  11  50  129  199 | 1 (reference)  2.04 (0.82-5.07)  3.10 (1.45-6.61)  9.57 (4.56-20.08)  <0.001  1.81 (1.55-2.11) |

^a^Analyses were adjusted for age (years) and sex, not for alcohol consumption; smoking aspects were also not mutually adjusted for.

^b^OCC: oral cavity cancer; OHPC: oro-/hypopharyngeal cancer; LC: laryngeal cancer.

^c^Tests for dose-response trends were assessed by fitting ordinal variables as continuous terms in the Cox proportional hazards model.

^d^Proportional hazards assumption was possibly violated for the model, and there was a statistically significant interaction of the exposure variable with time.

^e^Proportional hazards assumption was possibly violated for the exposure variable, and there was a statistically significant interaction with time.

Table A2. Associations (multivariable^a^ adjusted incidence RRs) between cigarette smoking and risk of subtypes of head-neck cancer, with (mutual) adjustment for smoking aspects; Netherlands Cohort Study (NLCS), 1986 - 2003

|  |  | **Subcohort** |  | **Head-neck cancer cases** | | | | | | | | | | |
| --- | --- | --- | --- | --- | --- | --- | --- | --- | --- | --- | --- | --- | --- | --- |
|  |  |  |  | **Overall** | |  | **Subtypes** | | | | | | | |
|  |  |  |  |  | |  | OCC^b^ | |  | OHPC^b^ | |  | LC^b^ | |
|  | **Categorical median** | **Person time at risk (years)** |  | **No. of cases** | **RR**  **(95 CI%)** |  | **No. of cases** | **RR**  **(95 CI%)** |  | **No. of cases** | **RR**  **(95 CI%)** |  | **No. of cases** | **RR**  **(95 CI%)** |
| **Cigarette smoking status, adjusted for frequency (*N*/day) and duration (years) of cigarette smoking** | | | | | | | | | | | | | | |
| Never smokers  Former smokers  Current smokers  *P* for trend^c^ |  | 25051  22644  16657 |  | 44  110  241 | 1 (reference)  1.78 (1.18-2.70)  4.05 (2.74-6.01)  <0.001 |  | 29  24  57 | 1 (reference)  0.86 (0.43-1.73)  2.40 (1.35-4.26)  0.003 |  | 6  22  55 | 1 (reference)  3.22 (1.15-9.01)  9.18 (3.57-23.58)  <0.001 |  | 9  64  126 | 1 (reference)  3.01 (1.41-6.42)  5.62 (2.62-12.03)  <0.001 |
| **Frequency of cigarette smoking (*N*/day), adjusted for cigarette smoking status (non-current/current) and duration (years)** | | | | | | | | | | | | | | |
| Never smokers  >0 to <20  ≥20  *P* for trend  Continuous, 10 cigarettes/  day increments | 0  10  20 | 25051  24787  14514  64352 |  | 44  155  196  395 | 1 (reference)  1.40 (0.91-2.15)  2.62 (1.72-4.00)  <0.001  1.34 (1.23-1.47) |  | 29  38  43  110 | 1 (reference)  0.69 (0.33-1.44)  1.29 (0.64-2.59)  0.11  1.32 (1.12-1.56)^d^ |  | 6  30  47  83 | 1 (reference)  2.28 (0.80-6.45)  5.64 (1.99-15.97)  <0.001  1.58 (1.36-1.83) |  | 9  85  105  199 | 1 (reference)  2.41 (1.11-5.22)  4.08 (1.89-8.80)  <0.001  1.26 (1.12-1.40) |
| **Duration of cigarette smoking (years), adjusted for cigarette smoking status (non-current/current) and frequency (*N*/day)** | | | | | | | | | | | | | | |
| Never smokers  >0 to <20  20 to <40  ≥40  *P* for trend  Continuous, 10 years  increments | 0  13  30  43 | 25051  7433  18999  12868  64352 |  | 44  20  105  226  395 | 1 (reference)  1.08 (0.61-1.90)  1.53 (1.00-2.33)^e^  2.78 (1.70-4.53)  <0.001  1.30 (1.17-1.45)^f^ |  | 29  4  30  47  110 | 1 (reference)  0.42 (0.14-1.29)  0.85 (0.43-1.68)  1.18 (0.48-2.94)  0.62  1.05 (0.87-1.27) |  | 6  5  25  47  83 | 1 (reference)  2.28 (0.64-8.06)  2.80 (0.99-7.91)  4.54 (1.43-14.39)  0.009  1.39 (1.11-1.74) |  | 9  11  50  129  199 | 1 (reference)  1.95 (0.78-4.88)  2.44 (1.13-5.25)  5.12 (2.25-11.65)  <0.001  1.51 (1.26-1.80) |

^a^Analyses were adjusted for age (years) and sex, not for alcohol consumption; analyses of cigarette smoking frequency and duration were adjusted for current cigarette smoking; cigarette smoking frequency and duration were also mutually adjusted for (continuous; centered) in analyses.

^b^OCC: oral cavity cancer; OHPC: oro-/hypopharyngeal cancer; LC: laryngeal cancer.

^c^Tests for dose-response trends were assessed by fitting ordinal variables as continuous terms in the Cox proportional hazards model.

^d^Proportional hazards assumption was possibly violated for the exposure variable, and there was a statistically significant interaction with time.

^e^*P*<0.05.

^f^Proportional hazards assumption was possibly violated for the model, and there was a statistically significant interaction of the exposure variable with time.
